# Analysis and/or Interpretation in Neurophysiology? A Transatlantic Discussion Between F. J. J. Buytendijk and K. S. Lashley, 1929–1932

**DOI:** 10.1007/s10739-022-09680-x

**Published:** 2022-06-09

**Authors:** Julia Gruevska

**Affiliations:** Faculty of Biological Sciences, Institute of Zoology and Evolutionary Research, History and Philosophy of Natural Sciences, Friedrich-Schiller-Universität Jena, Ernst-Haeckel-Haus, Berggasse 7, 07745 Jena, Germany

**Keywords:** Neurophysiology, Behavioral Sciences, Intelligence, Philosophical anthropology, Epistemology, Interpretation, Biophilosophy

## Abstract

In the interwar period, biologists employed a diverse set of holistic approaches that were connected to different research methodologies. Against this background, this article explores attempts in the 1920s and 1930s to negotiate quantitative and qualitative methods in the field of neurophysiology. It focuses on the work of two scientists on different sides of the Atlantic: the Dutch animal psychologist and physiologist Frederik J.J. Buytendijk and the American neuropsychologist Karl S. Lashley, specifically analyzing their critical correspondence, 1929–1932, on the problems surrounding the term *intelligence.* It discusses the inexplicable anomalies in neurophysiology as well as the reliability of quantitative and qualitative methods. While in his laboratory work Lashley adhered to a strictly analytic approach, Buytendijk tried to combine quantitative methods with phenomenological and hermeneutical approaches. The starting point of their discussion is Lashley’s monograph on *Brain Mechanisms and Intelligence* (1929) and the rat experiments discussed therein. Buytendijk questioned the viability of the maze-learning method and the use of statistics to test intelligence in animals; he reproduced Lashley’s experiments and then confronted Lashley with his critical findings. In addition to elucidating this exchange, this paper will, more generally, shed light on the nature of the disagreements and shared assumptions prevalent among interwar neurophysiologists. In turn, it contributes to historiographical debates on localization and functionalism and the discrepancy between analytic (quantitative) and interpretative (qualitative) approaches.

## Introduction

In the interwar period, there was not yet an overwhelming methodological consensus in neurophysiology; debates between researchers were oriented towards gaining such a consensus. Experimental psychology was still a young discipline. Although it had made striking advances and behaviorism had become firmly established (Mack, Lück, and Renner 2014; Mills 2000; Galliker 2007; Buckley 1989), the question of the most suitable method for investigating neurological processes was still open. Holistic, vitalist, and organicist approaches were discussed alongside mechanistic and behavioristic methods (Baedke 2019; Peterson 2017; Nicholson and Gawne 2015; Allen 2005). Discussions in the field of brain- and neurophysiology were especially centered on the issue of *functionalism* and the so-called *localization theory*. Although the question of where abilities were localized in the brain was widely recognized as a key research field— for instance, in the works of Marie-Jean-Pierre Flourens and Franz Joseph Gall — it was taken up with renewed vigor in holistic approaches (Folzenlogen and Ormond 2019; Guenther 2015; Pearce 2009; Finger 2000; Hagner 1995; Breidbach 1995; Harrington 1989; Finger and Stein 1982; Young 1970).^1^ Important representatives of these new perspectives were the Dutch physiologist and animal psychologist Frederik J. J. Buytendijk (1887–1974) and the American neuropsychologist Karl S. Lashley (1890–1958).

When Buytendijk read Lashley’s *Brain Mechanisms and Intelligence* in 1929, he partly agreed with and was partly skeptical of Lashley’s assertions. While he was somewhat convinced by the quantitative results of a rat experiment Lashley had conducted and discussed in the book, Buytendijk found the concrete modalities of the experimental setting problematic and considered Lashley’s interpretations of the results to be overly simplistic. Buytendijk was curious about whether he would obtain the same results himself and so he replicated Lashley’s experiment. His aim was not to discredit Lashley, but rather to open a discussion of a novel method in neurophysiological research.^2^

Lashley, professor of psychology at the University of Chicago and the president of the American Psychological Association in 1929, remained chiefly interested in the specific question of whether the brain could be divided into regions and whether each of these could be seen to have a particular function or ability (Weidman 1994, 1996; Dewsbury 2002). His approach to this topic consisted not only in collecting a tremendous amount of data, conducting statistical calculations, and carrying out numerous experiments, but also in combining holistic and behaviorist methods.^3^ However, Lashley did not consider the term *holism* to be applicable to any kind of subject or method of investigation but only to the brain as a complete organ (Harrington 1996; Ash 1995).

At the beginning of the twentieth century, holistic methods, in one way or another, still largely stemmed from the Aristotelian conviction that *the whole is more than the sum of its parts*. The *more* thus transcended the individual components. A whole is an entity in itself, and an entity for itself, and therefore not just a phenomenological appearance, but an ontological existence. However, because he located the whole not in the context of the entire organism but only in one organ, Lashley misunderstood or ignored the standard philosophical definition of holism. On the other hand, Buytendijk adopted the *philosophical* view of holism, as he stated (Buytendijk 1929). Accordingly, the experimenter should examine the individual subject in its entirety and not only the brain. It becomes apparent in this terminological difference that the fields of physiology sometimes assigned different meanings to the same concept. This made communication complicated, but also stimulating and fruitful.

In the case discussed in this paper, Lashley conducted experiments on healthy rats and rats with different cortical lesions and tested their ability to learn using mazes. This ability was, in Lashley’s view, the determinant of intelligence. He concluded from his results that there is no clear empirical support for the localization thesis in neurobiology and found evidence that it is not specific regions of the brain, but rather the whole mass of the brain tissue, that influences the behavior of the animals (Lashley 1929; see Finger 1994, p. 59; Hergenhahn 2004, p. 607).^4^ However, Buytendijk, who was the head of one of the largest physiological laboratories in Europe from 1925 to 1946, questioned whether insights on learning abilities derived from maze tests could really be used as the basis for claims about intelligence, as Lashley had supposed. Buytendijk considered intelligence to be a qualitative trait irreducible to quantitative extrapolations about a *brain mechanism*. Moreover, Buytendijk noticed inconsistencies that made him suspicious and eventually led him to try to replicate Lashley’s experiments in his own laboratory in 1931. He subsequently published a lengthy article on his results entitled *An Experimental Investigation into the Influence of Cortical Lesions on the Behavior of Rats* (1932), and laid out his concerns to the US-American psychologist in a number of letters. However, Lashley was aware of the conceptual imprecisions and shortcomings inherent in his interpretation of phenomena. This motivated his correspondence with European colleagues like Buytendijk.^5^

This paper takes as the starting point the discussion between these two scientists to develop a better understanding of the complex negotiations within interwar neurophysiology. In what follows, I first present an outline of Buytendijk’s biography as well as his theoretical and experimental approach to living beings. This is necessary because he is a largely forgotten figure in international interwar physiology and psychology. As a physiologist inspired by German philosophical thought (especially phenomenology and hermeneutics), his approach to the discipline of biology was neither vitalist nor mechanist nor organicist. He saw himself instead as an *anthropological physiologist*, to use his self-description from the 1960s (Buytendijk 1965; see also Fagot-Largeault 2009; Dehue 1995; Dekkers 1995). The following section discusses Lashley’s experimental program and rat experiments, which Buytendijk had questioned and at least partly rejected. Then I examine Buytendijk’s criticism of reductionism, drawing on his reflections on quantitative experimental methodologies, and discuss how he set out to repeat Lashley’s experiment in his own laboratory. In my analysis of their correspondence, I foreground Buytendijk’s critique of both Lashley’s understanding of the term *intelligence* and his “straightforward” use of statistics (Dewsbury 2002, p. 226). I then reconstruct how Buytendijk’s views on the methodological preconditions of experimental research arose from his phenomenological and hermeneutical approach. I conclude by reflecting more generally on the complex scientific negotiations we find in interwar neurophysiology. In the case of Buytendijk and Lashley, it is possible to see how diverse research positions in neurophysiology, and the theoretical and methodological differences between them, were negotiated. Their mutual skepticism comes to the fore in their attempts to conduct and discuss the same experiments. Ultimately, this article shows how scientific cultures clash in a multilayered discourse. Consideration of this case can therefore provide more general insight into the theoretical and practical challenges and the complexity of biology in the interwar years.

## Buytendijk’s Integrated Physiological Approach Towards Living Beings

Buytendijk, who studied medicine in Amsterdam, specialized in animal physiology and worked at various internationally renowned laboratories, such as Anton Dohrn’s Zoological Station in Naples (1906), and with Charles S. Sherrington, John N. Langley, and Archibald V. Hill in the UK (1912). The beginnings of Buytendijk’s zoological research lie in marine biology. His first publications as a student in 1909 were on the results of his experiments with *Sipunculus nudus*, an unsegmented marine worm that lives in subtidal sand. He studied sharks and gas exchange in the blood of cold-blooded animals. During the First World War, Buytendijk worked as a zoologist at the University of Amsterdam and in the psychiatric ward at the Valeriusplein Hospital in Amsterdam. After he completed his PhD at the Rijks-Universiteit in Utrecht under Hendrik Zwaardemaaker on habit formation in animals (*Proeven over gewoontevorming bij dieren*) in 1918, he became a full professor for physiology in Amsterdam, where he devoted his work to the border between biology, psychology, and physiology.

Although he was especially interested in questions of general biology, Buytendijk also conducted specific experiments on sensory perception and instinctive behavior. He therefore often reproduced the experiments of leading international animal psychologists like Wolfgang Köhler (1887–1967) and Robert Yerkes (1876–1956). Buytendijk studied the complexity of the senses in a wide variety of animals, from daphnia (Buytendijk 1918a) to dogs (Buytendijk 1924) to monkeys (Buytendijk 1921). In his experimental works on the complex relations between the environment, experience, and learning, Buytendijk did not regard animals as stimulus-reaction-machines where single stimuli bring about single reactions. Rather, his experiments revealed the epistemic limitations of overly simplified and reductionist methods and experimental settings. In Buytendijk’s view, these reductionist methodological patterns could only yield a simplified knowledge of organisms and their relation to the environment. That is why he conducted his experiments in large settings designed to reflect the animals’ natural habitat (Gruevska 2019a, b).

The most important finding Buytendijk made through his experiments during his Amsterdam period was that animals attach meanings (*Bedeutung*) to some stimuli and that these meanings can change over time. Stimuli that the animal initially found uninteresting, such as inanimate objects, can arouse its interest (and vice versa) when the animal’s psyche undergoes a sudden change, for example, from hungry to full. These can be primarily tested and observed with respect to the phenomena of pleasure (through food intake) and pain (through electric shocks). But other gradual experimental changes also revealed more complex mental processes and actions. For example, some stimuli had a more purposeful meaning (*zweckmäßige Bedeutung*) for some animals than others. Not all stimuli had the same meaning for all animals, or at least their reactions were not of the same intensity.

Some of Buytendijk’s experiments, such as tests on toads that showed the complexities of sensory perceptions and how they are brought together, left a strong lasting impression on him and intensified his interest in philosophy (Gruevska 2018). In experiments with the species *Bufo vulgaris* and *Bufo calamita*, Buytendijk decided to use both qualitative and quantitative analyses to study the instinctive return movements of toads to their nesting sites (Buytendijk 1918b, c). In this experimental setting, the animals could directly perceive the hatching angle during the experiment. At first, he used the quantitative parameters of duration and behavior to analyze habit formation. This showed that the times toads spent traveling the paths decreased significantly, even though the animals did not choose a shorter path, because the animals no longer hesitated in taking the path they had learned. Finally, these experiments studied the influences of various perceptual elements in learning in a way that also involved the experimenter. For example, when the animals had already learned to traverse an open space, the box was then turned around, meaning that they no longer walked away from the experimenter but toward him. Turning out the lamps above the apparatus also caused disturbance. This showed, according to Buytendijk, that the animals’ experience could only be applied to familiar, contextual situations. Initially, any environmental changes resulted in disturbances in the animals’ actions, but the animals quickly learned to follow the intended path. This changed as soon as another disturbance was introduced into the box. Nevertheless, Buytendijk stated that learned content also plays a biologically expedient role for the animals. Although animal actions are modified in habit formation, this always occurs in connection with innate instincts. Buytendijk concluded from these observations that competence in habit formation (learning processes) should be conceived as a kind of instinct that goes hand-in-hand with the animals’ other instincts (Buytendijk 1918b, c).

Unlike alternative explanations that explained these instinctive movements in terms of an associative relation between stimulus and response, Buytendijk recognized an experiential influence on the nest-seeking instinct in a change in the understanding of total sensory impressions. He contended that it is not possible to single out individual sensory impressions and individual stimuli taken in by the senses and to establish them in their individual relations to one another. Instead, multiple factors play important roles in constituting animals’ responses to their environment.

Already in these early experiments on sensory physiology in Amsterdam, Buytendijk recognized the epistemological problems associated with reductionist approaches to research. He also noted how findings from animal experiments could be transferred in an interesting way to philosophical anthropological problems (human-environment relations). In 1923, Buytendijk read the book *Die Einheit der Sinne. Grundlinien einer Ästhesiologie des Geistes* (*The Unity of the Senses: Basic Lines of an Aesthesiology of the Spirit*) by the German philosopher Helmuth Plessner (1892–1985), which dealt with problems of sensory perception from a philosophical point of view (Plessner 1923). Buytendijk was impressed by Plessner’s work, which had numerous similarities to his own findings, and he invited Plessner to work with him in Amsterdam. Plessner, who was himself a trained zoologist, accepted Buytendijks invitation. The two conducted experiments and published a joint paper on the perception and interpretation of mimic expressions (Buytendijks and Plessner [1925] 1980). Buytendijk and Plessner remained lifelong friends and worked together during the Second World War, when Plessner had to leave Germany because of his Jewish origins (Gruevska 2021; Becker 2015; Fischer 2009; Dietze 2006). As will be shown later, the Buytendijk and Plessner collaboration was a key influence on Buytendijk’s view of the epistemological questions in natural sciences that were central to his debate with Lashley.

In 1925, Buytendijk was appointed director of the Physiological Institute at the Rijksuniversiteit in Groningen. Founded in 1911, this was the first institute in the Netherlands to describe itself as exclusively a physiological institute. Profiting from the tireless efforts of the previous director Hartog J. Hamburger (1859–1924) and from Buytendijk’s direction, the Groningen Physiological Institute became one of the most influential international research centers of its time, not least owing to its excellent laboratory. Buytendijk continued Hamburger’s pioneering work in analytically- and experimentally-oriented physical chemistry. Like many other contemporary physiologists, Hamburger did not regard animal psychology as a subdiscipline of the science of organic functions. Buytendijk, however, had a different ethos regarding the scope of the discipline and used different conceptual frameworks (de Haan 1925). He focused more strongly on the investigated object as a living subject and reflected on the role and responsibility that the investigator assumes in the laboratory setting (Thines and Zayan 1975). This focus went hand-in-hand with his favorable contrast of the phenomenological, understanding method with the causal-explanatory one. Rather than engaging in an analytical examination of specific constitutive components, Buytendijk set out to inquire about the more general properties of the animal psyche and their relation to the animal’s physiological constitution. Such generalist, philosophical inclinations were also manifest in Buytendijk’s understanding of the epistemological underpinnings of the explanatory approach of science and concomitant concrete experimental work (Gruevska 2018).^6^

One of the key beliefs of Buytendijk and other early twentieth-century psychologists and physiologists was that a human science would only be possible if it considered the human being as a whole (Prick 1974). This approach meant combatting both the tendency toward a one-sided reductionism and vitalist approaches to humans. Buytendijk confronted these positions with a philosophically-informed, two-way (*integrated*) research framework that took both viewpoints into account. He drew on experimental observations and the idea that bodily existence is a *holistic experience* that transcends the linear sequences of physiological states. If, for example, one studies a child learning to walk, one can see the child trying to move his or her *whole* body and not just tensing or relaxing different parts of muscles or nerves (Buytendijk and Plessner [1925] 1980). Buytendijk maintained that this basic holistic stance could be transferred to various other problem areas.

## The Need for a Completely New Brain Physiology

Buytendijk’s philosophical position led him to reject Cartesian dualism and to advocate the hybridization of physiology and psychology – a theoretical stance common in early twentieth-century German-speaking philosophical anthropology.^7^ Helmuth Plessner, who together with Max Scheler (1874–1928) pioneered philosophical anthropology in interwar Germany, corresponded and worked intensively with Buytendijk (Buytendijk and Plessner 1925, 1935). Plessner characterized Buytendijk’s physiology as situated in the “tension between the Galilean and Aristotelian concepts of epistemology” (Plessner 1957, p. 388).^8^ In the 1920s, Buytendijk was regularly invited to Germany and particularly to Cologne to give talks on the notion of the organism and other biological topics. He also published articles in philosophical journals (among others, Buytendijk [1925] 1980; 1928; [1929] 1958; 1941; 1942). As a member of the German-speaking intellectual and academic establishment, Buytendijk came into close contact with phenomenologists and philosophers of different persuasions – not the least at Scheler’s soirées. And while their conceptions of living beings strongly influenced him, they, in turn, admired his scientific expertise. It seemed fruitful to Buytendijk to combine philosophical and scientific approaches.

In his view, the organism was a phenomenon of life: an entity that is constituted by *psyche* and *physis.* This is also where the hermeneutic and phenomenological aspects of his work came to the fore (van Buuren 2018; Spiegelberg 1972). As he expressed it, “the phenomena of life can be understood both as action and as expression” (Buytendijk 1925, p. 18). Buytendijk explicitly linked this view to the humanist Wilhelm Dilthey’s (1833–1911) distinction between explaining and understanding. Dilthey argued that we explain *nature*, and therefore physis, yet we *understand* the psyche (Dilthey [1894] 1924, p. 144). In this account, life was seen as a “psycho-physical unity” (Dilthey [1894] 1924; Scheler [1913–1916] 2000; [1928] 2007, p. 157; Plessner [1928] 1975). In all, this meant that the life sciences needed both understanding and explanation. However, by integrating physiological and (anti-dualistic) philosophical viewpoints, Buytendijk was not breaking completely new ground in neurobiology (Grene 1974; Gruevska 2019a, b). In trying to explicitly integrate his theories into physiological praxis, Buytendijk was following a research framework that became increasingly influential in brain studies, a field that had already been established in the 1880s and that interlinked anatomy and psychology, embryology and evolutionary theory, as well as physiology and philosophy (Stahnisch 2020; Borck 1999, p. 146).

With this transdisciplinary approach, Buytendijk was able to introduce epistemic questions into his institutional setting and experimental work in a targeted and constructive manner. This methodology reinforced his thesis that any reductionism, simplification, or one-sided theoretical approach – the behaviorist research program, for instance – proved inadequate when it came to analyzing the living. Instead, Buytendijk stressed that living beings were constituted not only by chain reflex mechanisms but by numerous different parameters in their interactions with the natural environment (Gruevska 2019a, c). This breadth and diversity necessitated a much wider theoretical and methodological scope, and Buytendijk accordingly attempted to integrate animal psychology more closely into physiology.

In his 1925 inaugural lecture *Über das Verstehen der Lebenserscheinungen* (*On the Understanding of Phenomena of Life*) at the University in Groningen, Buytendijk stated that his own research program would put less emphasis on “one of the many certainly very interesting individual problems [of his] subject” and rather focus on the general question of “whether the methodological rules of physiology promote an increase in knowledge” (Buytendijk 1925, p. 10). He was convinced that his philosophically grounded experimental approach would allow for more promising investigations of the “phenomena of life” while also establishing a novel and more reflected research method in physiology. Buytendijk held that it was his “duty” and he had “every right” to question the limited methods of physiology with regard to their philosophical and epistemological parameters (Buytendijk 1925, p. 10).

Buytendijk’s critique was grounded in a detailed analysis of the theoretical and methodological assumptions dominant in his field. He remarked critically that induction had become the only accepted research method in physiology. He considered this as having resulted from an increasingly radical separation of empiricism and philosophy. As a result of this development, the method of induction had become restricted to such a degree that it was now virtually coextensive with the analytical method. However, Buytendijk also noticed a counter-development: he believed that an increasing number of scientists wished to open up their field and research practices again (Buytendijk 1925, p. 21). He saw this not only as desirable and necessary but also highly advantageous. Discussing previous materialistic approaches such as those of Frans Cornelis Donders (1818–1889), Charles Darwin (1809–1882), Theodor Schwann (1810–1882), Jakob Moleschott (1822–1893), and Jacques Loeb (1859–1924), Buytendijk pointed out that, although the conflict between these two research programs was an old and recurring problem, it was only recently that it had begun to be considered from a completely different perspective. “[N]ow that the question arises anew as to whether the living in its composite forms and functions perhaps possesses a multiplicity of original qualities that are destroyed by [reductionist] analysis” (Buytendijk 1925, p. 12). Buytendijk stressed that both the quantitative physiological details and the qualitative dimension that constituted the unity of the object under investigation must be taken into account. His critique did not aim at addressing isolated points or even at deconstructing empirically oriented scientific methods. Rather, he hoped to establish a more self-reflective research method and a “more intimate familiarity with the reality surrounding us” (Buytendijk 1925, p. 12). Buytendijk wanted, above all, to put a “stronger emphasis on ‘understanding’ alongside ‘explaining’” (Buytendijk 1925, p. 13; see also Feest 2010; Grene 1974).

In his 1929 essay *Zur Untersuchung des Wesenensunterschieds von Mensch und Tier* (*On the Investigation of the Essential Difference between Man and Animal*), Buytendijk positioned his research within the international biology of the time. He admitted that, although biology was dominated by the “analytical method and the classification of facts in the genetic evolutionist scheme,” a different research program was being established. Its method was the “direct observation of organic wholeness,” and it tried to gain deeper biological knowledge from a novel “theoretical foundation” (Buytendijk [1929] 1958, p. 21). Buytendijk correctly observed that there had been a trend in the first decades of the twentieth century that included attempts to point out the insufficiency of purely physicochemical explanations of phenomena of life (as in the work of Hans Driesch 1867–1941]); to re-discuss the notion of functionality (as John B. S. Haldane [1892–1964] had done); or highlight (as in Jakob von Uexküll’s work), the “basic logical errors and the experience-adverse structure of Darwinism” (Buytendijk [1929] 1958, p. 21). However, Buytendijk was not completely convinced by the new programs developed by Uexküll or the vitalists because they did not produce any valid results or insights. According to Buytendijk, they instead produced results only *ex negativo* and did not provide any positive solutions.^9^ Nonetheless, he acknowledged that the circumspect analysis of physiological experiments had produced the most valuable insights to date into the working of organic functions. Yet, at the same time, Buytendijk considered this analytical reductionist method to be unsuitable for the task of paramount importance to him: the study of organic life as a whole (Buytendijk [1929] 1958, p. 21).

While charting these general vitalist and holistic tendencies in interwar biology, Buytendijk remarked that brain researchers had also been discussing the supposed wholeness of the brain. This debate was centered on the concepts of localization and functionalism and whether there were specific regions in the brain that were responsible for specific behavioral abilities and functions. Buytendijk expressed critical reservations about this debate, stating that even those scholars who sought to study the overall function of the brain tended to apply the analytical method and, in most cases, only investigated its individual parts. He therefore argued that a “completely new brain physiology” was needed, claiming that the emergence of *Gestalt*-psychology had rendered the older concepts and methods obsolete (Buytendijk [1929] 1958, p. 23). This became clearer when the view that physiological phenomena were to be considered as (only) a totality of parts had been proved incorrect. Yet Buytendijk believed that, given the lack of high-profile institutions committed to biological research, it was going to be difficult to solve this problem in the foreseeable future. He complained that “it is actually a testimony of powerlessness of science that it accepts the separation of physiology and psychology in principle and thus cannot recognize the given natural psycho-physical unity of the body as its research object” (Buytendijk [1929] 1958, p. 23). He, however, was determined to change this situation.

## Buytendijk’s Replication of Lashley’s Experiment

Buytendijk saw an opportunity to begin a productive exchange with Lashley about the problems he explored, especially because they had different research approaches. Buytendijk was even determined to use Lashley’s “psychobiological” work as a starting point for redirecting brain research towards his integrative “phenomenological” approach (Weidman 1996). The core of this attempt was a detailed discussion of Lashley’s famous behavioral experiments on rats.

In the experiment in question, Lashley studied the learning behavior of rats by analyzing the neural mechanisms that formed part of the learning process. He examined 19 rats whose cortexes were injured ranging between the degree of between 14 and 50%. He investigated whether they were still able to remember the paths leading out of the labyrinth, which they had learned when they were healthy, after parts of their cortex were removed. This experimental setup was to reveal which parts of the cortex were or were not involved in memory formation and storage. If a certain area of the brain could be shown to be responsible for memory, this could be evidence for the validity of the then widely discussed localization thesis concerning the brain. However, Lashley’s experiments did not establish this. He argued instead that the experiments showed that the functions of the brain must be distributed across the whole brain. In fact, these experiments showed that it did not matter which brain areas were removed but rather how much of the total brain mass was eliminated.

During his experiments, Lashley also noticed that while some of the skills the rats had learned before the operation were initially no longer present, the animals were able to re-learn them quickly. This led him to conceive his theory of *equipotentiality*. He argued that it was possible for still-intact brain areas to help in performing those functions, such as speech in the case of humans, for which other now-destroyed brain areas had previously been responsible (Lashley 1929). Such a plasticity in the whole brain seemed to be revealed, for instance, in aphasia patients who had suffered a stroke. Lashley thought he could prove that human brains, too, were plastic by extrapolating from the tests and experiments Kurt Goldstein had conducted with brain-damaged soldiers and other patients (Goldstein and Gelb 1918). Lashley also considered his findings to be compatible with those of his colleague and close friend Shepherd Ivory Franz (1874–1933), who had examined the behavior of brain-damaged patients in Washington, DC and carried out experiments on brain-operated monkeys (Franz and Lashley 1917; see Beach 1961). The specific nature of such a takeover of functions, however, was still anything but clear to Lashley, not the least because “[w]e know little of the interrelations of the two hemispheres” (Lashley 1929, p. xi).

In order to understand Buytendijk’s approach to Lashley’s work, we need to take a closer look at Lashley’s study. In the first chapter of *Brain Mechanisms and Intelligence*, entitled “Theories and Problems,” Lashley claimed: “To measure intelligence and to deduce from the resets of measurement some conception of the nature of the function, the problem of the physiological basis of complex adaptive behavior has been largely ignored” (Lashley 1929, p. 1). Lashley intended to address this problem. In his view, it was necessary to examine intelligence from a physiological perspective because many theories of intelligence had already been provided from a psychological perspective. In addition, he considered the available theoretical approaches to proceed mainly from metaphysical assumptions that were not scientifically sound. In reviewing influential theories of his time, ranging from those developed in the works of William McDougall (1871–1938), Charles Spearman (1863–1945), Edward Thorndike (1874–1949), *Gestalt* theory, as well as older models such as Flourens’s, Lashley flatly concluded that they were all insufficient (Lashley 1929, pp. 1–15).

In Lashley’s opinion, the behaviorist view of reflex theory was also inadequate. On that point, he and Buytendijk were actually in agreement. Dewsbury summarized Lashley’s position as follows: “Lashley argued against a psychology based on chain reflexes and in favor of central regulation” (2000, p. 269). However, notwithstanding their shared rejection of chain reflex models and the localization theory, Buytendijk was critical of Lashley’s attempt to ground the study of intelligence on his experiments. Buytendijk did not so much criticize behaviorism as a blinkered methodology; rather, he suggested that its reliance on statistics is not always helpful, especially when it came to qualitive questions (such as intelligence). He even went so far as to claim that Lashley had “no right” to talk about *intelligence* at all (Buytendijk 1932, p. 371). In this regard, Buytendijk referred to Hunter and Randolph’s article “Further Studies on the Reliability of the Maze with Respect to Rats and Humans,” maintaining that it “demonstrated that the same animal which is slower than another animal in learning a certain labyrinth often learns quicker after some time when the experiment is repeated” (Buytendijk 1932, pp. 23, 371; Hunter and Randolph 1924).

Unlike Buytendijk, Lashley was not convinced by that article, not least because Hunter and Randolph only “dealt with groups of normal adult animals which probably include a relatively small range of variation and which show insignificant correlation between the various constants used measure individual differences” (Lashley 1929, p. 20). In contrast, Buytendijk stressed that the “correlation calculus of such double experiments show that the labyrinth method is a fully inefficient means of measuring intelligence” (Buytendijk 1932, p. 327). To test this claim, he repeated Lashley’s experiment. However, his commitment to quantitative analytical methods meant that he would also endeavor to observe and interpret animal behavior. According to Buytendijk, examining the living primarily with the help of statistics is not enough: “I have therefore repeatedly urged that – besides the statistical method that is always applied by the American behaviorists – it is also essential to carefully observe the individual conduct of the animals” (Buytendijk 1932, p. 327).

**Fig. 1:**
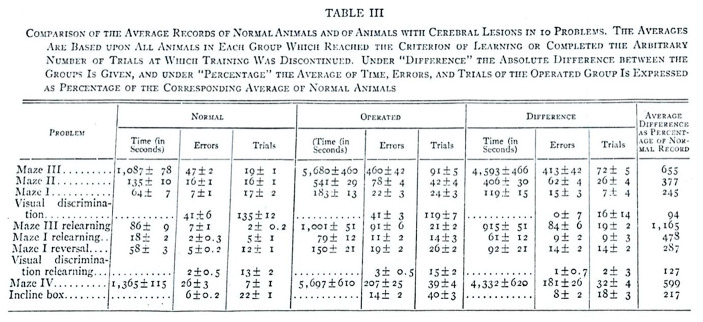
From Lashley 1929, p. 42

Lashley did not publish individual protocols for his experiments, but he did summarize the most important data in tables and diagrams. The diagrams show the individual lesions for each experiment arranged according to the size of the injury and without specifying the injured brain areas. In particular instances, however, these diagrams summarized a number of cases. “These have been constructed by superimposing the diagrams for the separate cases and blocking in either the areas common to all or the total area covered by all cases under consideration” (Lashley, p. 18). Lashley included in his measurements of maze-learning performance (and subsequent calculations of this) the three most important criteria for learning: total time, total trials, and total errors, which precede the achievement of an arbitrary number of error-free trials. For example, in pre- and post-operative retention tests, Lashley used either the individual recordings or the average learning scores of a group as a standard for comparison. In the operated group of mice, the score was determined as a percentage of the corresponding average for normal animals and then the average of these percentages was represented in a combined diagram depicting the above three criteria (for an example, see Fig. 1).

Because individual variations, both in the extent of the lesion and in the training records, deviated from the curve of normal distribution, Lashley used the *rank difference method*

**Fig. 2:**
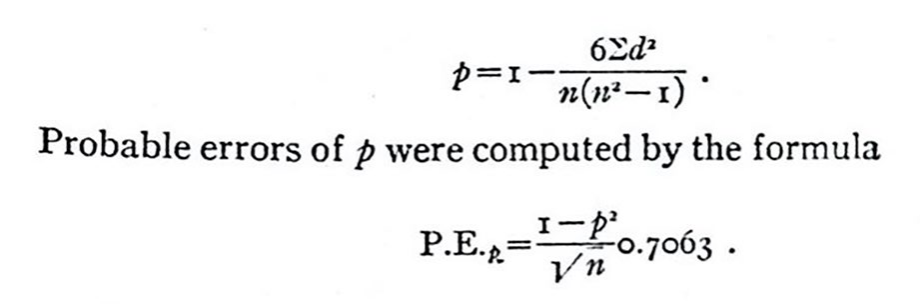
From Lashley^1929^, p. 19

– established by Spearman – to calculate and visualize the correlations (see Fig. 2).

The individual post-operative recordings for learning in mice were compared with the group average for the preparatory training recordings, given that Lashley had concluded that differences in the recordings of normal animals were largely due to chance and not characteristic of the individuals.

Buytendijk criticized Lashley’s statistical methods for their insufficient consideration of individual variations in learning and behavior. In Buytendijk’s experiments, a number of rats with lesions varying in their extent and cortical location were challenged to relearn the mazes they had learned before the surgery. At a later time (which depended on when the rats died, which could be hours, days, weeks, and even months after the experiment), the rats’ brains were autopsied. The study showed that all rats who had been operated on had had suffered severe losses in fine-motor coordination and were less adapted to their environmental surroundings. Buytendijk believed the latter claim to be vouchsafed by the rats’ reduced activity in the maze and their unnatural sensitivity, either “greatly intensified or greatly reduced” both to noise signals and in the interaction with the experimenter (Buytendijk 1932, p. 434). Buytendijk further noticed greater “lability of conduct, unrestrainedness (not adapted to the situation), impairment of the totality to be integrated by consecutive details of an action and reduced insight and reproduction of purposeful actions” (Buytendijk 1932, p. 434).

**Fig. 3:**
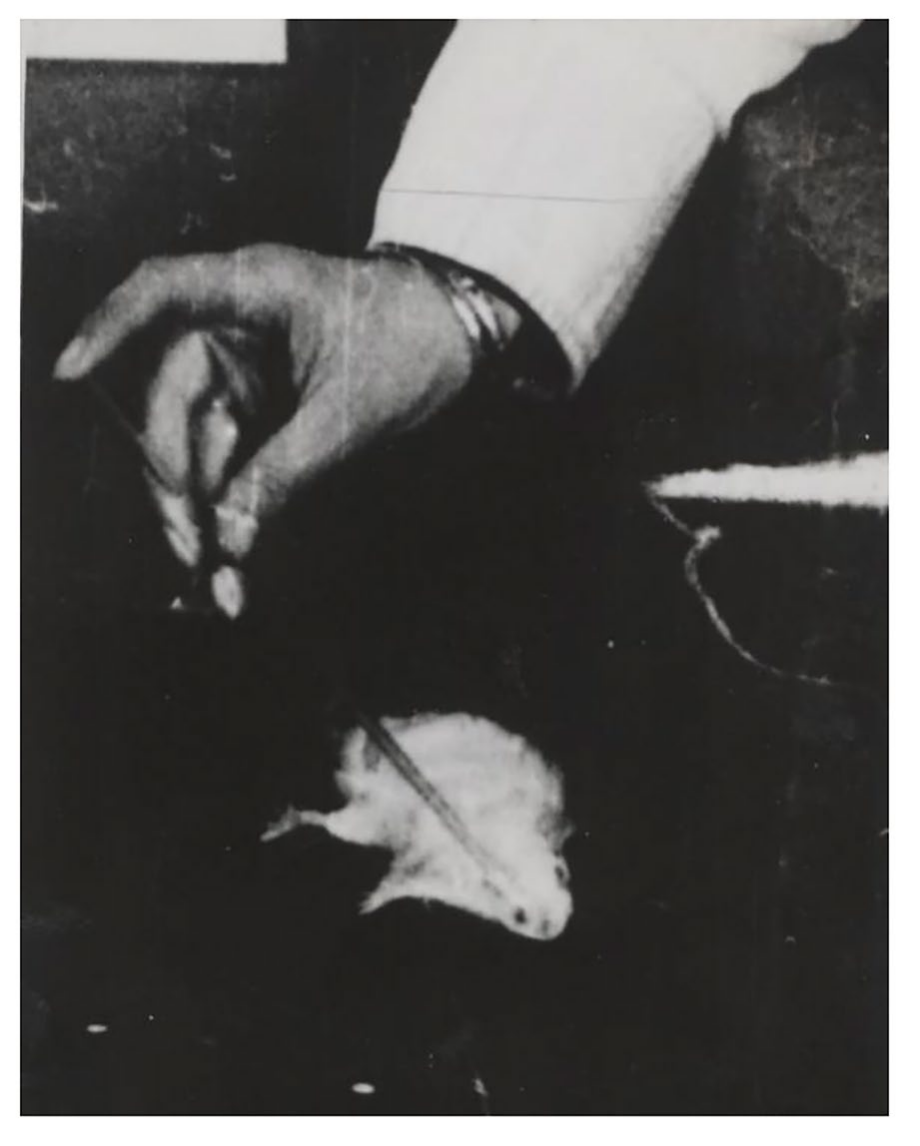
Abnormal behavior of rat No. 2. Rat lies on the side while drinking milk from a tube (Laboratory protocols, BUYT-1355, Buytendijk Archive, KDC)

**Fig. 4:**
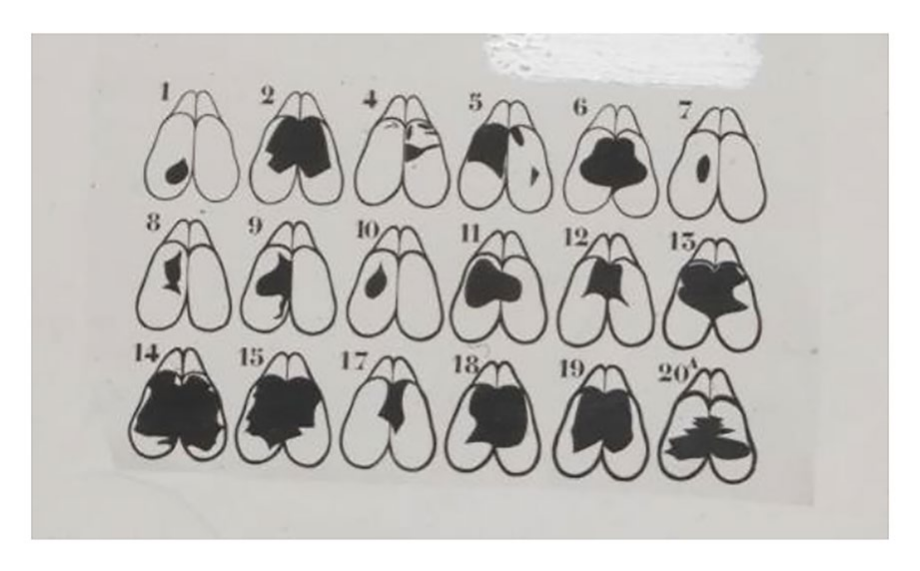
Cerebral lesions of the operated rats (Laboratory protocols, BUYT-1355, Buytendijk Archive, KDC)

Buytendijk and his research team noted in their experiment protocols that brain-operated rats exhibited anomalies in various actions. Buytendijk thought it impossible for research oriented by statistics to measure or even notice those anomalies. For example, some of the operated animals exhibited altered behavior regarding food ingestion, smelling, and general movement. In more complex experimental setups—for instance, when observing the rats’ drinking from glass pipettes—they showed anomalous postures and movements (Fig. 3). In this experiment, at least twenty rats with different degrees of lesions underwent operations on different parts of their brains (Fig. 4). A multitude of “normal” rats were used as a control group for behavioral comparison and the identification of anomalies. According to Buytendijk, these rats could not only be bred in large numbers, but the non-operated animals also showed an amazing consistency in behavior under the stable experiential conditions, thus facilitating behavioral comparisons.

While other critics, such as the psychologist James Papez (1883–1958), considered the rat, given its small brain, to be completely unsuitable as an experimental animal for complex behavioral and neurophysiological studies, Buytendijk merely warned against jumping to conclusions from the observations made.^10^ While recognizing that these experiments had yielded some interesting results, Buytendijk nevertheless noted that because of the size of the rat brain, it was impossible to make high-precision lesions in it. The use of thermocautery, for example, was particularly problematic for two reasons. First, due to the small brain size, it was not possible to surgically remove exactly (and exclusively) the intended parts and, second, the heat emanating from the thermocautery device could destroy parts of the brain tissue even when not coming in direct contact with it. Thus, Buytendijk, unlike Papez, did not directly criticize Lashley’s choice of animals but rather questioned the use of the thermocautery device in general. Its heat might disrupt blood circulation and could cause thrombosis in rats. According to Buytendijk, disturbances in blood circulation in the brain were not unlikely to cause the destruction of the surrounding ganglion cells. These side-effects could not be controlled and could lead to different behavioral patterns. What was more, the heat could directly cause irreparable damage in the ganglion cells, which affected the functional performance of neural regions. The latter problem is particularly severe because the functional disturbance in neurons could not be proven histologically. Buytendijk further claimed that “[c]auterization of tissue causes the formation of decomposition substances, which are cell-poisons, causing oedema in the adjoining tissue” among other things (Buytendijk 1932, p. 375). Similarly, Buytendijk observed in his experimental series that thermocautery often brought about difficulties stemming from its tendency to induce invisible injuries. Cold knife incisions were an alternative to thermocautery, yet they proved even more problematic because the recently-destroyed brain area was more likely to become inflamed and cause cognitive impairments, with some rats exhibiting “schizophrenia”-like symptoms.

In appraising Lashley’s methodological setting, Buytendijk came to two conclusions. First, given that the experimental object was a very small animal with a correspondingly small brain, determining the relation between injuries and possible behavioral changes following from them was particularly difficult (Buytendijk 1932, p. 376). This is why the experimenter must take into account the fact that “the cortical lesions in rats may be larger than the microscopic-anatomical examination reveals” (Buytendijk 1932, p. 376). Second, and related to this, the lack of precision in cauterization probably caused lesions to be more extensive and functionally damaging than they appeared. Buytendijk therefore warned against drawing hasty conclusions from these experiments: “It is very difficult, if not impossible to determine the relation between the destruction anatomically stated and the phenomena observed” (Buytendijk 1932, p. 376). In short, Buytendijk’s critique was that the method chosen was incapable of clearly connecting particular lesions to brain functions and behavior. In Buytendijk’s words, this experimental setup “renders any discussion of localization problems in these small animals illusive” (Buytendijk 1932, p. 376). Such a setup, that is, could neither clearly prove nor reject the localization theory of the brain. Nevertheless, Buytendijk admitted that such experimental studies were worth pursuing because the attempt to trace an animal’s operation-induced behavioral aberrations helped in gaining a better understanding of their general characteristics.

## Buytendijk’s Critique of Lashley’s Use of the Term *Intelligence*

Buytendijk did not primarily intend to verify or falsify the results of Lashley’s quantitative data collection in reproducing the rat experiments. He instead wanted to draw his own conclusions from them, that is, create his own phenomenological access to them so that he could assess whether Lashley’s interpretations were appropriate. As a philosophically informed animal psychologist, Buytendijk was chiefly concerned with the conceptual framework Lashley used, namely, that centered around the notion of intelligence. Lashley aimed to conceptualize and explain the rats’ behavior with regard to its supposed manifestation of, lack of, or degree of intelligence.

Lashley’s intelligence test built on both the earlier work of Edward Lee Thorndike, who had tried to develop a test that could be applied to several species, and on Charles Spearman’s conception of intelligence. Both had relied on the traditional distinction between sensory and associative brain cortexes. Intelligence was then considered a function of the strength of cortico-cortical connections, that is, the ability to form associations. However, smaller animals, which did not have a pronounced association cortex, were then thought to have only sensory abilities, which prevented them from perceiving their environment in the form of distinct objects. Moreover, such a one-sided conception did not allow for the study of complex social structures and behavior. Thorndike tried to overcome these obstacles by designing standardized test situations that could be used to study the speed, for instance, of learning mechanisms in cats and monkeys. Yet it proved to be difficult to classify animals by applying a simple, uniform learning test. Lashley, therefore, extended the test situation to include brain-operated animals as well. He developed his concept of intelligence mainly from Spearman, who had put forward a mathematical argument to establish a concept of intelligence with general validity (see also Fig. 2). To test intelligence—in this instance in humans—experimenters need only to investigate various kinds of logico-mathematical and verbal reasoning as well as memory strength. Based on this methodology, Spearman was certain that it would be possible to make valid and generalizable quantitative assertions about intelligence that were independent of different test weightings (Lashley 1929). Lashley sought to draw up a series of maze-solving tests for mice that would resemble such tests of human intelligence (see Diamond 1981).

For Lashley, the understanding of intelligence used in his maze tests had a firm grounding in physiology and neurology. Buytendijk (1932), for his part, did not consider the maze-learning method to be suitable for the examination of intelligence.^11^ As stated above, he thought it impossible to measure intelligence quantitatively, not the least because he still held to the eighteenth-century notion of intelligence as “mental ability, wisdom” derived from the Latin “intelligentia,” “insight, cognitive faculty” (Toepfer 2011, p. 215). He deemed the “double-path method,” which he developed with Werner Fischel, to be more suitable for the study of intelligence of this kind (Buytendijk and Fischel 1931). The double path was structured as follows: The rats first learned a long route back to their nest, and afterwards they were shown a shorter route. Whenever a rat chose the shorter one after memorizing these two non-intersecting paths, Buytendijk concluded that it chose that route out of “insight” (Buytendijk 1932, p. 427).

Generally speaking, faster and slower learning processes could be observed in both operated and non-operated rats. Whereas Lashley linked intelligence directly to learning, Buytendijk, noting that the experiment showed operated rats to be capable of learning more quickly than normal rats, advised that “we should be careful” when “assuming that a choice-reaction, whether concerning a direction or a visual perception, is a sign of ‘intelligence.’” (Buytendijk 1932, p. 405). In Buytendijk’s view, the attempt to determine intelligence in animals by extrapolating from maze-learning was premised on faulty methodological assumptions. The same held for conclusions drawn from animal behavior involving relearning or learning to move in another direction after they had first been taught a different way: “If we compare the ‘learning in the other direction’ of the normal and the operated rats, we are struck by a great difference” (Buytendijk 1932, p. 407). After about five to twenty-five attempts, nine out of ten of the normal rats succeeded in following the newly learned path without any error, there remained one rat that did not. Rat no. 4 needed more than forty attempts to complete the new way without any mistakes. After that, however, the animal succeeded sixty times in a row without any mistakes. “Therefore, although apparently the relearning took place more slowly than in other animals, the conduct of this rat is not less ‘intelligent’” (Buytendijk 1932, p. 407).

Since he had found a great variability in the statistical data obtained from observing the animals’ behavior (especially regarding those rats that had lost more than 25% of their cortexes), Buytendijk deemed it inadmissible and negligent to calculate and work using average values. He argued such data did not allow the derivation of any serious scientific claims. Moreover, Buytendijk found fault with the small number of test animals used: “In view of this variability in the operated animals and the comparatively small number of test animals it would be inadmissible to calculate average figures.” Therefore “[a]ll these facts prove once more that it is necessary to study the animals individually during the whole period of the experiments and also to record all peculiar features in their manner of conduct in addition to the number of experiments, mistakes and time” (Buytendijk 1932, pp. 410–411).

While Buytendijk was reluctant to conceive of the rats’ behavior in Lashley’s experiments in terms of intelligence, he did believe that a certain kind of intelligence had to be attributed to those animals. In fact, he noticed intelligent behavior even in animals that had undergone surgery. But in contradistinction to Lashley, Buytendijk understood intelligence as referring exclusively to individual behavioral acts. To him, such individual acts were, strictly speaking, the opposite of learned behavior, given his definition of intelligence, even in animals, as a kind of insight or understanding. The results of a second experiment, for instance, seemed to him to indicate that the animal’s concrete behavior was “very strikingly ‘intelligent’” because the “rat runs halfway in the wrong direction, then stops suddenly, turns around and runs directly and without hesitation to the right end of the cross-path” (Buytendijk 1932, p. 411). It was thus “evident that rat Nr 8 acts with comprehension” (Buytendijk 1932, p. 412). Whereas Buytendijk noted that other animals, did not exhibit such “insight” and instead acted “more mechanically,” he was nonetheless convinced that many, if not most, showed insight (Buytendijk 1932, p. 413). The rats understood that they now had to choose a different path. Ultimately, however, he could not judge the different rats’ behavioral choices to be subject to variations and fluctuations between wrong and right to an unacceptably high degree. Therefore, Buytendijk stated that no conclusions about the rats’ intelligence could be drawn from registering the number of mistakes made and the amount of time they spent when trying to fulfill their tasks, and that the same held for any of the other numerical data obtained from the re-learning experiment. Instead, the “observation of each animal separately is … necessary” (Buytendijk 1932, p. 415).

For Buytendijk, animal intelligence referred to acquired behavior that is based on the perception of the relationship between the context of a particular situation and the available patterns of movement. Animals do not learn stereotypical movements but always patterns of movement (“senso-motoric categories”). According to Buytendijk, insight is a physical, senso-motor dependent relationship that is to some extent applicable to all animal species capable of adapting to new situations. If such insight occurs in an animal, then one can also speak of intelligence or intelligent behavior. Insight is thus a sudden recognition of the context of a situation and action together with an available activity familiar through experience. In other words, intelligence is finding – without judgment – the appropriate tool for the right situation and using it in the appropriate way. By discussing various examples, Buytendijk tried to show that the kinds of intelligence exhibited by different species can hardly be compared in a satisfactory manner. Additionally, he claimed that it is always difficult to decide whether it is by trial and error or by its actual practical intelligence that an animal learns to use purposefully the means necessary to reach its goal (Buytendijk 1957).^12^

In addition, Buytendijk argued that, within the animal kingdom, intelligence differs only in degree, not in kind. Each species has its own species-specific practical intelligence. Numerous experiments on animals’ learning ability conducted in his institute tried to show that it is not habit but the sensual experience of the situation’s structure that determines the behavior of an “intelligent” animal (see de Haan 1926). In contrast, after brain injuries, habitual behavior comes to the surface. Moreover, there are general species-specific limits to intelligence, but there are also individual differences (lazy and curious animals).

This shows that, while the concept of intelligence played a special role for Buytendijk in many ways, he treated it with caution. He thought that using terms imprecisely could lead to making faulty assertions and arguments, which could ultimately lead to reaching false conclusions about animal psychology as a whole. It is against this background that Buytendijk took issue with Lashley’s use of the concept of intelligence and his use of quantitative methods to make sense of phenomena that, for Buytendijk, were only accessible by the observation and interpretation of the experimenter. He leveled his harshest criticism at Lashley’s all too careless establishment of causal connections between the animals’ experimentally induced anatomical injuries and subsequent abnormal behavior. In many cases, he concluded, there were simply no causal relations. While some animals exhibited abnormal behavior, the autopsies after their death did not show this to be linked to the injuries induced. When Buytendijk made this observation, he attempted to provide possible explanations for why this was the case. Lashley, however, ignored this issue. Buytendijk warned that “we should not forget that the intact animal is not a reflex-machine and that the quantitative results are only means to express qualitative phenomena” (Buytendijk 1932, p. 407).

## Exchanges on the Experiments Between Buytendijk and Lashley, 1930–1932

Although Lashley and Buytendijk’s respective results were largely similar, their approaches to the interpretation of these results were strikingly different. To Buytendijk, Lashley’s conclusions still seemed questionable, mainly because microscopic-anatomical examinations conducted during the autopsies revealed that often the nature of the brain-operated rats’ lesions did not correspond to their behavioral abnormalities. How, under these circumstances, could one make any authoritative statements about anything observed? How could one include unknown agencies in such explanations? Regarding these problems, Buytendijk was thus concerned about drawing hasty conclusions or even about ignorance on Lashley’s part. Falsely judging an animal to be lacking in intelligence would not only do an injustice to the object of investigation but also ultimately lead to distortions in the field of comparative psychology as a whole, since subsequent research would then proceed on the basis of prior misinterpretations.

Buytendijk wrote a letter to Lashley to discuss these problems, seemingly during the period when he was conducting his own series of rat experiments (rat no. 1 was operated on April 1930 and died in August 1930).^13^ In his response, Lashley cordially welcomed Buytendijk’s attempt to initiate a conversation. He wrote to him that he had been following his work “with interest for years”^14^ and that he was highly interested in Buytendijk’s opinions about the problems that, at that time, concerned them both and the discipline of neurophysiology. Apparently, Buytendijk had begun the correspondence by bringing up the symptoms of schizophrenia visible in some of the rats, asking Lashley if he had had made similar observations and, if so, what his explanation was. Buytendijk was certainly referring to the behavior of rat no. 2, which had made a strong impression on him because its behavior was strikingly different to that of the other rats. Lashley affirmed that he had made many similar observations, “especially after operations with a knife.” But he had no sure explanation of how and why this behavior occurred. He believed that it could be a consequence of “intracranial pressure or to metabolic disturbances.” It also seems Buytendijk had asked Lashley if he had developed a theory of neural organization. In his reply, Lashley said that he had not, explaining that it was “first necessary to establish the facts more certainly.”^15^ Clearly, this conversation revolved around the uncertainties that troubled both scientists, yet it also seems to indicate that Lashley was not as plagued by these problems to the same extent as Buytendijk.

The second of Lashley’s letters in Buytendijk’s archive is an explicit response to Buytendijk’s 1932 article on his replication of the rat experiment. It appears that Buytendijk had sent a copy of his paper to Lashley, who answered with a four-page letter, apologizing for its length. Interestingly, Lashley did not address Buytendijk’s criticism of the term *intelligence* but rather moved the discussion in another direction. He only discussed findings where he believed he had reliable data and statistics and was able to provide explicit evidence. He conceded that Buytendijk raised “a number of very important questions, on some of which we seem to have significant data. The most puzzling question of all is that of individual differences in behavior after nearly identical lesions.” Lashley stated he had come across several such cases. In a series of experiments on “detail vision” carried out on fifty rats, 49 rats had lost their power of detail vision due to the same injury, yet one rat was still able to distinguish visual patterns. In his letter, Lashley professed that he was unable to account for this. However, his statements show that, unlike Buytendijk, he thought it was better not to focus on such exceptions because “(un)known disturbing agents could only obscure but not produce such a consistent relationship” between physiological paradigms.

Responding to Buytendijk’s criticism of the labyrinth tests, in the letter Lashley defended them as “reliable if properly used,” yet failed once again to address the question and problem of intelligence. Regarding the maze tests, he responded to Buytendijk’s objections as well as to those of Hunter and Randolph (1924) by insisting on the reliability of coefficients, “ranging from 0.85 to 0.95 for operated animals in learning of two mazes.” Lashley went on to say that he considered some of Buytendijk’s criticisms – among others, the list of many of the possible reasons for “non-focal disturbance” – to be irrelevant and unimportant (Buytendijk 1932, pp. 374–375).^16^

## The Experimenter’s Need for Interpretation and Reflection

When Buytendijk replicated Lashley’s series of experiments, he recognized and critically reflected on the fact that, despite his using much the same terminology, their respective setups were still not identical. One reason for this was that the animals used were different individuals with different “personalities.” Buytendijk considered this an important detail, which many animal psychologists, especially mechanistically oriented ones, did not take into account. Further, Buytendijk was convinced that every scientist framed the experiment according to their individual methodological beliefs and unconscious assumptions. This included distinct approaches to the modalities of observing and understanding the animal and its behavior. Buytendijk thought that in order to produce accurate experimental results, the researcher must become familiar with the animals through long-term observation, thus learning to understand them and interpret their concrete movements. Only by developing this kind of familiarity was it possible to differentiate between the deviation and the norm.

Buytendijk illustrated this phenomenological stance and its epistemic consequences by discussing a striking incident. One day after lesions (though no deep wounds) had been surgically induced in the right and left frontal cortex of rat no. 2, it suffered from hallucinations and developed “acute schizophrenia”—it snatched for nonexistent objects in the air and even appeared to attack and fight an imaginary opponent. The animal psychologists also observed that, similar to humans with mental disorders, the brain-damaged animal behaved less abnormally in open spacious rooms. Three hours after the surgery, the “schizophrenic” rat no. 2 started to behave like a normal rat again.^17^ “When placed on the floor, the animal runs as far as the corner of the room, remains there for some time and then starts running spontaneously, mousing and erecting itself against the wall. After having been going to and fro for some time along the wall, covering a short distance only, the rat withdraws somewhat further from this spot, then returns and recommences running to and fro.”^18^ The behavior of the rat recently operated on did not differ from that of non-operated rats, as became apparent when their paths and actions were compared (see Gruevska 2019c; Van der Veer 2000). However, while the behavior was superficially identical, a closer scrutiny of the actions of the individual animal revealed that the movements and physical execution of rat no. 2 deviated from the norm. Its movements looked, as Buytendijk wrote, less *strukturiert* (structured), using the only German word in the test protocol. That is, they did not transition smoothly into one another.

Such fine-grained observations about the concrete setting of his experiment led Buytendijk to cast doubt on the general validity of the methods of quantitative analysis. He discovered that neither the path the rat followed nor the speed with which it moved was in any way different from those of a healthy rat. But, as the person conducting the experiment, he was nonetheless able to notice that there were in fact differences. From a phenomenological point of view, the rat’s behavior did not look like that of a healthy animal. Finally, he stressed the tremendous responsibility of the experimenter for the outcome his chosen method yielded: they would develop sophisticated skills during long-term experiments, closely observing the individual animal to understand its behavior. Accordingly, Buytendijk advocated extending the neurophysiological and analytical approaches, suggesting they required that the scientist familiarize themselves with the singular qualities of the research object. Given that Lashley addressed none of those epistemological questions in his monograph or his letters, it seems safe to say that they were of little, if any, concern to him.

## Conclusions

All too often in the history of biology, it has been assumed that one would be hard-pressed to find anything more multifaceted and contentious than the conflict between neo-vitalists or holists and mechanists raging at the beginning of the twentieth century. Surprisingly, however, detail-oriented historical studies provide a more nuanced picture of interactions between the alternative perspectives. They illuminate certain commonalities as well as somewhat inconspicuous theoretical struggles between mechanists and vitalists. In the midst of fundamental debates in interwar brain research, contemporaneous researchers developed a variety of views that tried to link mechanistic perspectives and methodologies with approaches eager to address the phenomenologically accessible properties of living beings, such as their agency and decision-making, in non-reductive ways. Debates about functionalism and behaviorism reflected those attempts (Nicholson and Dupré 2018; Nicholson and Gawne 2015; Oyama 2010; Allen 2005). There were also approaches, typically overlooked, that cannot be located neatly at or between the classic poles of mechanistic (including analytical reductionist methodologies) and vitalist-oriented accounts of the teleological agency of organisms. These were developed on a transnational scale in the course of debates about neural functionalism, the localization thesis, and reductionist behaviorism. While American and European scholars agreed on the necessity of both abandoning purely functionalist approaches and better integrating psychology and physiology, they often disagreed about the positive characteristics of the holistically oriented alternative method to be developed. While some investigators considered analytical, quantitative approaches as suitable for studying the limits of the localization thesis (Lashley, Franz), others (Buytendijk, Straus, Grünbaum) wanted to bring about a more thoroughly phenomenological shift in comparative neurophysiology, one that would move the discipline towards including qualitative behavioral properties of individual organisms.

By contrast, this paper suggests that it was their shared interest in rather opaque problem areas that brought scientists with seemingly incompatible starting assumptions together instead of driving them even further apart. Interestingly, cooperation was desired rather than rejected, and disagreements between two parties could be discussed productively. Whether such cooperation can ultimately be considered successful is a different question. In the case discussed in this paper, this must be answered in the negative. Thus, it cannot be said that the mechanistically oriented biosciences, such as behaviorism, followed a linear, successful path. Nor can it be claimed that they dominated the behavioral biosciences from the very beginning: there were other serious theoretical frameworks in biology in circulation alongside that approach.

This paper has drawn attention to the fact that the developments in interwar biology cannot be represented as monolithic: the historical documents tell a very different story. It should be recognized that this was a particularly heterogeneous period, an epistemic battlefield, where the search for “truth” involved researchers considering views that were incongruent with their own ideas, yet that they nevertheless took into account in order to find at least an approximate solution to certain problems. This period was also characterized by openness and exchange, far more than it might initially appear in retrospect (Leahey 1992). Further, this paper has shown that the debates on brain physiology and comparative psychology in the interwar period were both complicated and challenging. Indeed, a continuous debate on the nature of scientific evidence took place between and among analytical physiologists and interpretative psychologists. From the case presented in this paper, we can conclude that not only the interpretative or vitalist but also the behaviorist approaches harbored self-critical and self-reflective scientists within their ranks. The interwar period proved to be a turbulent time in the history of biology, one in which many uncertainties prevailed. Interwar biology was still in the process of clearing the haze. Above all, a commonly accepted theoretical framework was not available that could pave a clear path to guide researchers through these difficult times. Instead, theory was in a mode of intensive discourse – between diverse views and across borders. Finally, this analysis demonstrates that it is worthwhile and necessary to inquire further into the diverse theoretical and methodological stances in early twentieth-century neurological research. There are many other obscure connections between scientists that are yet to be illuminated.

## Footnotes

1 During World War I, the famous studies of Kurt Goldstein and Adhémar Gelb (1918) had shown that abilities like speaking could be relearned even when the cortical part of the brain, responsible for language, was injured or missing.

2 See letters from Karl S. Lashley to Frederik J. J. Buytendijk, undated, BUYT-164, Archive F.J.J. Buytendijk, 211 BUYT, Katholiek Documentatie Centrum (KDC) Nijmegen. Erasmuslaan 36, 6525 GG Nijmegen, Netherlands; hereafter BUYT-XY, Buytendijk Archive, KDC.

3 Lashley was enthusiastic about experiments and about German philosophy and culture (Roofe 1970). This proclivity, as the historian of science Nadine Weidman pointed out, was not merely an arbitrary private pleasure on his part; rather, it strongly influenced Lashley’s political opinions, the effects of which need to be taken into account in discussing his life and experimental work (Weidman 1994, 1996, 2002; Dewsbury 2002). Racist assumptions permeate Lashley’s work: he was a supporter (not financially or practically, but ideologically) of German nationalism and apartheid (Weidman 1994, 1996, 2002; Dewsbury 2002). Against this backdrop, his concept of intelligence in particular needs to be considered critically. Although this important issue deserves to be studied in detail, here my main aim is discrepancies between these two representatives of a strict analytic approach versus a philosophical-epistemological approach to the same experiment.

4 Buytendijk and Lashley both took positions that were, or would become, highly influential in this debate. Lashley’s results were still discussed ambivalently up to the late 1970s (Thomas and Knowles Weir 1975).

5 Another important German psychologist for Lashley was Kurt Goldstein (1878–1965). The psychologist and Goldstein student Marianne Simmel remarked of Lashley and Goldstein’s friendship: “Although their conversation was always in English, Goldstein used to say about Lashley he was ‘so European’ and that was why one could discuss problems with him so well” (Simmel 1968, p. 8). This statement obviously did not apply to Buytendijk’s experience with Lashley, as will be shown below.

6 For instance, Buytendijk was also preoccupied with the theory of the environment developed by Jakob von Uexküll, a biotheoretician of great significance for German philosophical anthropology (Gruevska 2019c; Köchy 2015; Becker 2015; Bühler 2004) but of lesser interest for physiological research (Mildenberger and Herrmann 2014, p. 314). Buytendijk agreed with Uexküll’s assertion that the animal forms a unity with its environment. It was on this basis that Buytendijk questioned and criticized behaviorist and mechanistic approaches that examined animals and their behavior in restricted experimental settings such as T-mazes (Gruevska 2019b). Buytendijk kept trying to find and formulate the ideal approach for investigating living beings. In correspondence, Uexküll once described Buytendijk as still being a “strict logician” (letter from J. Uexküll to Buytendijk, 11/8/1921, BUYT-344, Buytendijk Archive, KDC) and was surprised that Buytendijk supported his theory because most analytic-minded scientists rejected his research as unscientific and therefore unreliable. See also Mildenberger and Herrmann (2014, p. 314).

7 Buytendijk actively drew on philosophical anthropology, which is also sometimes referred to as “biophilosophy” (Köchy 2015) or as the “philosophy of biology/philosophical biology” Grene 1968, 1974; Grene and Depew 2004), as a field that took great interest in biological theories.

8 Unless otherwise indicated, all translations from Plessner’s and Buytendijk’s German works are by the author.

9 Note, however, that both the vitalistic and holistic positions (especially in the cases of Driesch, Haldane, and von Uexküll) stimulated, and were closely linked to, empirical investigations (see Baedke 2019; Peterson 2017; Köchy 2015).

10 On the question of the laboratory rat as an experimental organism, see especially Clause (1993).

11 It is striking that throughout the whole manuscript, Buytendijk used the term “intelligence” in quotation marks.

12 Buytendijk distinguished between the intelligence of animals and humans. In animals, insight into objective relations makes it possible for them to perceive and act on the relationship between tool and purpose without, of course, understanding this relationship as humans do (Buytendijk 1957). In addition, Buytendijk considered animals, unlike humans, to be incapable of actions based in reason, that is, actions informed by a theoretical conceptualization of the situation. In his view, it is always possible for humans to find potentially new solutions to practical problems (Buytendijk 1957).

13 Unfortunately, Buytendijk’s letters to Lashley cannot be found in either scientist’s archive or estate. This summary of the correspondence is therefore based on Lashley’s letters in response, which are in the Buytendijk Archive (KDC). While one-sided, it is nonetheless possible to follow their arguments given that Lashley was responding to the questions raised by Buytendijk’s 1932 article. It is thus central to the analysis presented here.

14 Lashley to Buytendijk, undated, BUYT-22-5, Buytendijk Archive, KDC.

15 Lashley to Buytendijk, undated, BUYT-22-5, Buytendijk Archive, KDC.

16 Lashley to Buytendijk, undated, BUYT-164, Buytendijk Archive, KDC. Lashley countered that the pressure created in some of the rats’ brain regions is probably responsible for these problems. Citing his own experimental work, he also remarked that, contrary to Buytendijk’s claim that damaged cells remain unabsorbed, he has found that they do, in fact, disappear after six months: “I cannot believe therefore that any significant areas of the cortex or thalamus could remain non-functional but normal in appearance after the 6 to 9 monthe [sic] during which most of our cases are kept under observation.”

17 However, Buytendijk noticed that the lesions in the central nervous system of these animals healed faster and more “holistically” than was the case for people with cerebral disorders.

18 Laboratory protocols, p. 14, Foto’s betreffende diverse proefnemingen, BUYT‐ 1355, Buytendijk Archive, KDC.

## Archival materials

All archival material is cited by historical date and folder number from the Katholiek Documentatie Centrum [**KDC**] located at Nijmegen. Erasmuslaan 36, 6525 GG Nijmegen, Netherlands.
